# Zinner Syndrome in Young Adult Males: A Case Series and Literature Review

**DOI:** 10.7759/cureus.59552

**Published:** 2024-05-02

**Authors:** Praveen K Sharma, Polaka Yashaswinii, Arun Aram, Karpagam RK, Sakthi Ganesh Subramonian

**Affiliations:** 1 Radiodiagnosis, Saveetha Medical College and Hospital, Saveetha Institute of Medical and Technical Sciences (SIMATS) Saveetha University, Chennai, IND

**Keywords:** infertility, wolffian ducts, urogenital abnormalities, seminal vesicles, congenital renal agenesis

## Abstract

Zinner syndrome (ZS) is a highly uncommon congenital or developmental urogenital anomaly characterized by the triumvirate of unilateral renal agenesis or dysplasia, ipsilateral ejaculatory duct obstruction, and ipsilateral seminal vesicle cyst. We present three cases of ZS in a 21-year-old male, a 20-year-old male, and a 24-year-old male. The diagnostic evaluation revealed unilateral renal agenesis associated with hypertrophy of the ipsilateral seminal vesicle with cystic changes on investigation by ultrasonography (USG), computed tomography (CT), and magnetic resonance imaging (MRI). The patients underwent surgical management, resulting in symptom resolution and enhanced quality of life. This case report highlights the diagnostic challenges, management options, and long-term outcomes for patients with ZS.

## Introduction

Zinner syndrome (ZS) is an uncommon congenital developmental male urogenital anomaly affecting the distal Wolffian or mesonephric duct between the fourth and thirteenth weeks of gestation [1]. Incomplete migration of the ureteric bud from the proximal mesonephric duct fails, and its failure of fusion with the metanephros, which plays a vital role in the differentiation of the metanephric blastema, and atresia of the ejaculatory duct lead to a triad of unilateral and ipsilateral ejaculatory duct obstruction, ipsilateral renal agenesis (IRA), and ipsilateral seminal vesicle cyst. Zinner described this syndrome for the first time in 1914 [2]. ZS often is noticeable in the second or fifth decade of life and can cause serious side effects, most notably infertility. Other manifestations of ZS include urgency, dysuria, pollakiuria, prostatism, epididymitis, perineal discomfort, painful ejaculation, and hematospermia [3].

## Case presentation

Case 1

A 21-year-old male presented with a history of recurrent lower urinary tract infections associated with intermittent hematospermia for six months prior to presentation. The patient had no issues of infertility. Physical examination revealed no abnormalities, and routine laboratory investigations were within normal limits, including semen analysis. Urine cultures repeatedly demonstrated *Escherichia coli* infection, prompting further investigation.

**Figure 1:**
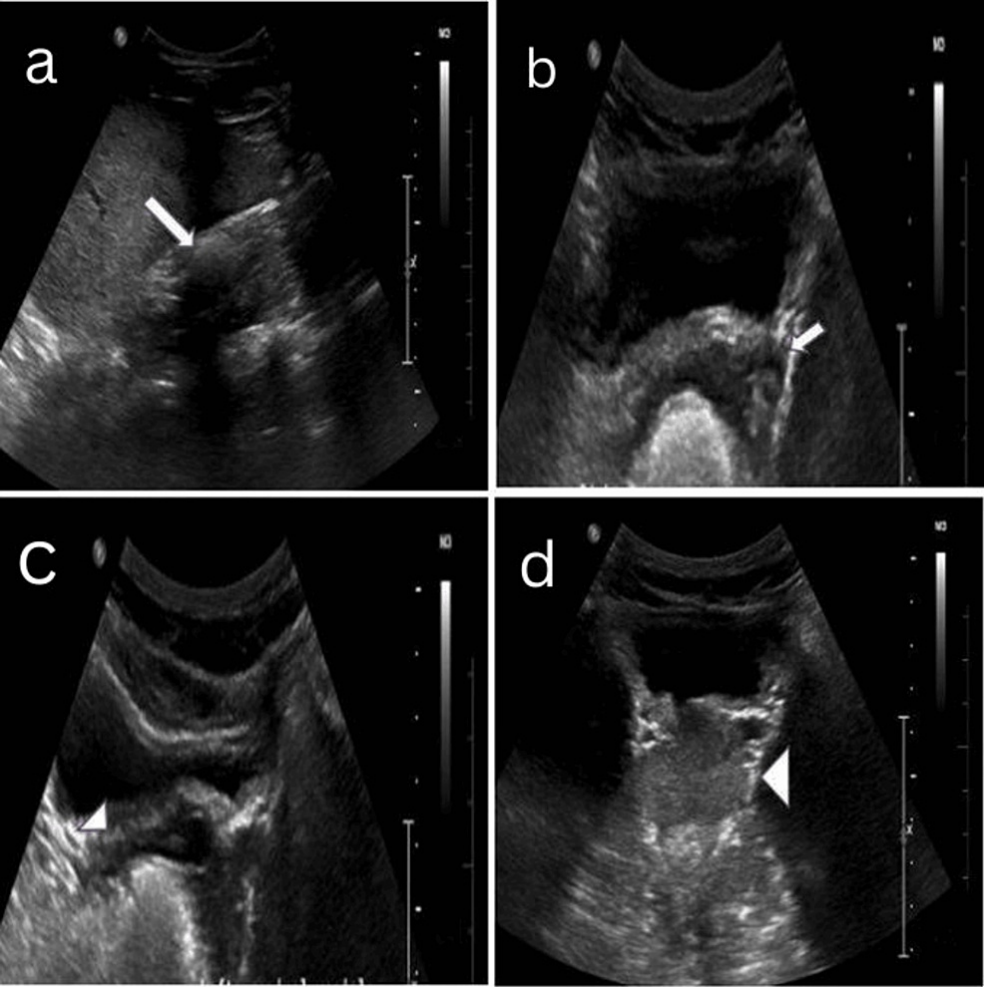
A 21-year-old male presented with a history of recurrent lower urinary tract infections associated with intermittent hematospermia over the past six months. (a) USG of the abdomen (longitudinal and transverse views) shows non-visualization (absence/agenesis) of the left kidney in the left lumbar region (large thick white arrow); (b) left seminal vesicle was enlarged with cystic changes (small thick white arrow); (c) right seminal vesicle was normal (small white arrowhead); and (d) prostate was normal (large white arrowhead). USG: Ultrasonography

Ultrasonography (USG) of the abdomen showed non-visualization of the left kidney in the left lumbar region with an enlarged ipsilateral seminal vesicle of size ~ 44 x 33 mm (length x width) with cystic changes. In contrast, the right seminal vesicle appeared normal. The prostate appeared normal (Figure 1a-1d).

 

**Figure 2:**
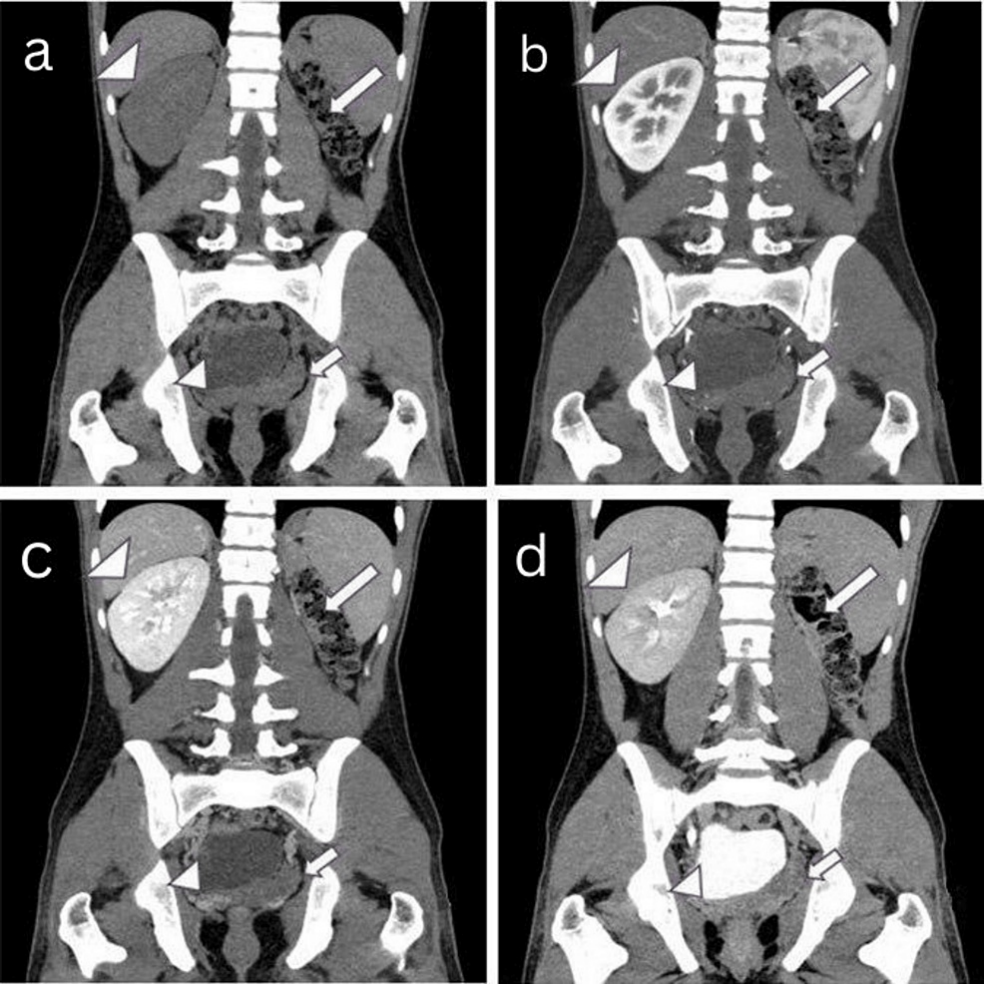
A 21-year-old male presented with a history of recurrent lower urinary tract infections associated with intermittent hematospermia over the past six months. (a) Plain CT of the abdomen in coronal view and (b,c,d) CECT abdomen in coronal view (cortico-medullary, nephrographic, and excretory phases) showed non-visualization (absence/agenesis) of the left kidney in the left lumbar region (large thick white arrow), left seminal vesicle was enlarged with cystic changes (small thick white arrow), right kidney was normal (large white arrowhead), and right seminal vesicle was normal (small white arrowhead). CT: computed tomography CECT: contrast-enhanced computed tomography

Computed tomography (CT) of the abdomen (plain and contrast-enhanced in the corticomedullary, nephrographic, and excretory phases) showed non-visualization of the left kidney and renal vessels in the left lumbar region with an enlarged ipsilateral seminal vesicle of size ~44 x 33 mm (length x width) with non-enhancing cystic changes. In contrast, the right kidney and right seminal vesicle appeared normal (Figure 2a-2d).

 

**Figure 3:**
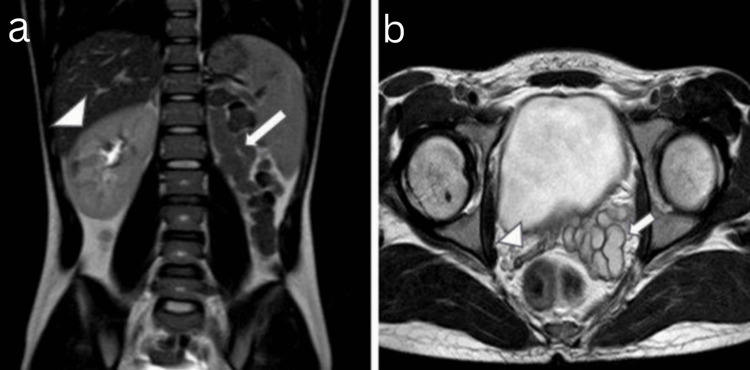
A 21-year-old male presented with a history of recurrent lower urinary tract infections associated with intermittent hematospermia over the past six months. MRI of the abdomen : (a) T2W coronal showed non-visualization (absence/agenesis) of the left kidney in the left lumbar region (large thick white arrow), right kidney was normal (large white arrowhead); (b) T2W axial showed enlarged left seminal vesicle with cystic changes (small thick white arrow), and the right seminal vesicle appeared normal (small white arrowhead). MRI: magnetic resonance imaging T2W: T2 weighted

Magnetic resonance imaging (MRI) of the abdomen showed T2-weighted images with non-visualization of the left kidney and renal vessels in the left lumbar region, along with an enlarged ipsilateral seminal vesicle of size ~44 x 33 mm (length x width) with cystic changes. In contrast, the right kidney and right seminal vesicle appeared normal (Figure 3a, 3b).

 

Based on the clinical and imaging findings, ZS was considered. After an interdisciplinary discussion involving urologists and reproductive specialists, it was decided to proceed with surgical excision of the seminal vesical cyst, and the patient was followed up 1 week post-op and subsequently at 2 months and 6 months. There were no post-operative complications at 1 week follow-up. There was an absence of urinary tract infections and hematospermia during further follow-up visits.

Case 2

A 20-year-old male presented with a history of recurrent lower urinary tract infections associated with prostatism, urinary urgency, frequency, dysuria, pelvic/perineal discomfort, and scrotal pain for three months prior to presentation. The patient did not have any infertility issues however; semen analysis was done and the results proved to be normal. Physical examination revealed no abnormalities and routine laboratory investigations were within normal limits. Urine cultures repeatedly demonstrated *Staphylococcus saprophyticus* infection, prompting further investigation. 

**Figure 4:**
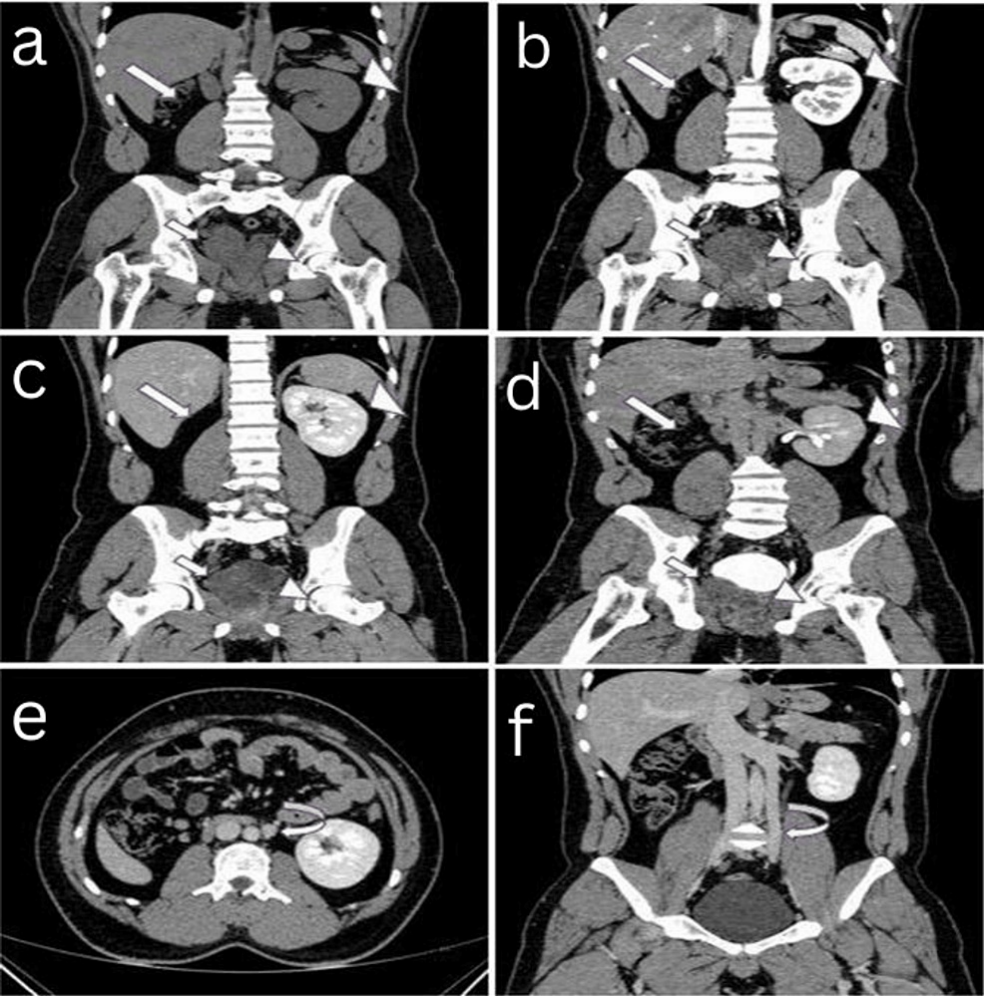
A 20-year-old male presented with a history of recurrent lower urinary tract infections associated with prostatism, urinary urgency, frequency, dysuria, pelvic/perineal discomfort, and scrotal pain over the past three months. (a) Plain CT of the abdomen in coronal view; (b-d) CECT abdomen in coronal view (cortico-medullary, nephrographic, and excretory phases) showed non-visualization (absence/agenesis) of the right kidney in the right lumbar region (large thick white arrow), right seminal vesicle cyst (small thick white arrow), left kidney was normal (large white arrowhead), left seminal vesicle was normal (small white arrowhead); (e,f) CECT abdomen in axial and coronal views (nephrographic phase) showed duplicated inferior vena cava (curved white arrow). CT: computed tomography CECT: contrast-enhanced computed tomography

A computed tomographic (CT) scan of the abdomen (plain and contrast-enhanced in the cortico-medullary, nephrographic, and excretory phases) showed non-visualization of the right kidney and renal vessels in the right lumbar region with ipsilateral seminal vesicle cyst of size ~34 x 37 x 41 mm (AP x TR x CC), while left kidney and left seminal vesicle appeared normal and was associated with duplicated inferior vena cava (Figure 4a-4f).

 

**Figure 5:**
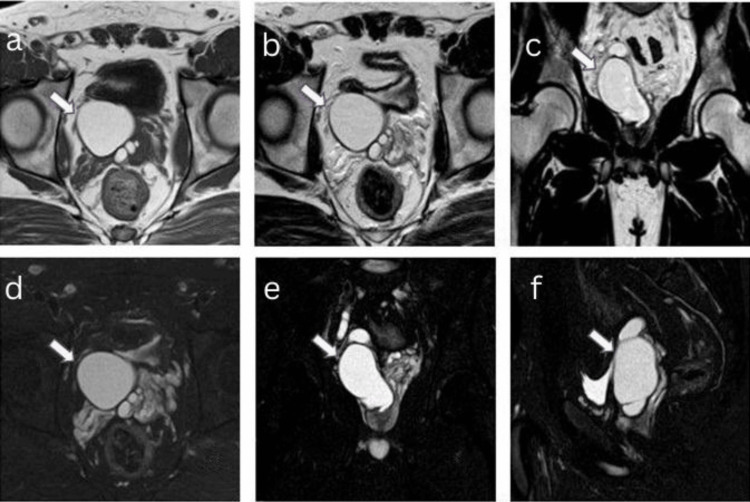
A 20-year-old male presented with a history of recurrent lower urinary tract infections associated with prostatism, urinary urgency, frequency, dysuria, pelvic/perineal discomfort, and scrotal pain over the past three months. MRI of the abdomen: (a) T1 axial - focal hyperintense; (b) T2W axial showed hyperintense uni-loculated cystic lesion in the right seminal vesicle, central homogeneity (likely proteinaceous content), peripheral smooth thin margin; (c) T2 VISTA coronal; (d) SPAIR axial; (e) SPAIR coronal; (f) SPAIR sagittal - hyperintense signal. MRI: magnetic resonance imaging SPAIR: spectral attenuated inversion recovery T1W: T1 weighted T2W: T2 weighted VISTA: volume isotropic turbo spin echo acquisition

Magnetic resonance imaging (MRI) of the abdomen showed T1-weighted images with focal hyperintense and T2-weighted images with hyperintense uni-loculated cystic lesion in the right seminal vesicle, measuring approximately 34 x 37 x 41 mm (AP x TR x CC). The lesion exhibited central homogeneous signal intensity, likely indicating proteinaceous content, with a peripheral smooth thin margin. Additionally, T2 volume isotropic turbo spin echo acquisition (VISTA) images showed hyperintensity, spectral attenuated inversion recovery (SPAIR) images showed hyperintensity, diffusion-weighted imaging (DWI) showed no reduced diffusivity, and gradient echo (GRE) showed no blooming. The right ejaculatory duct measured approximately 3.5 mm in diameter and appeared mildly dilated (Figure 5a-5f).

 

Based on the clinical and imaging findings, ZS was considered. After an interdisciplinary discussion involving urologists and reproductive specialists, it was decided to proceed with surgical excision of the seminal vesicle cyst to improve the patient's symptoms and urinary discomfort. Subsequently, during the six-month follow-up, there was an absence of prostatism, urinary urgency, frequency, dysuria, pelvic/perineal discomfort, and scrotal pain. The patient was advised a yearly follow-up to assess the durability of the surgical outcome and monitor for any signs of cyst recurrence or other complications.

Case 3

A 24-year-old male presented with a history of recurrent lower urinary tract infections associated with dysuria, frequency, perineal pain, and irritation following ejaculation for six months prior to presentation. The patient was not aware of any symptoms pertaining to infertility. Physical examination revealed no abnormalities, and routine laboratory investigations were within normal limits. Semen analysis, however, was found to be normal. Urine cultures repeatedly demonstrated *Escherichia coli* infection, prompting further investigation. 

**Figure 6:**
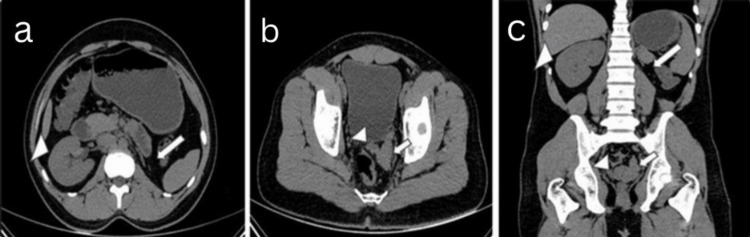
A 24-year-old male presented with a history of recurrent lower urinary tract infections associated with dysuria, frequency, perineal pain, and irritation following ejaculation over the past six months. Plain CT of the abdomen in (a,b) axial and (c) coronal views showed non-visualization (absence/agenesis) of the left kidney in the left lumbar region (large thick white arrow) and left seminal vesicle was enlarged with cystic changes (small thick white arrow), right kidney was normal (large white arrowhead), right seminal vesicle was normal (small white arrowhead). CT: computed tomography

Computed tomography (CT) of the abdomen (plain) showed non-visualization of the left kidney and left renal vessels in the left lumbar region, with an enlarged ipsilateral seminal vesicle with cystic changes. In contrast, the right kidney and right seminal vesicle appeared normal in size (Figure 6a-6c).

 

Based on the clinical and imaging findings, ZS was considered. After an interdisciplinary discussion involving urologists and reproductive specialists, it was decided to proceed with surgical excision of the seminal vesicle cyst. The patient underwent surgical management and was counseled for follow-up. However, the patient did not follow up with us post-operatively.

## Discussion

ZS is a rare urogenital tract anomaly characterized by ipsilateral renal agenesis or dysplasia, ejaculatory duct obstruction, and seminal vesicle cysts. This syndrome was first documented by Zinner in 1914. According to Sheih et al. [4], there were 13 cases of pelvic dilatations linked to ipsilateral renal agenesis, indicating a 0.00214% frequency. ZS is diagnosed between the second and fifth decades of life when the seminal vesicle cyst is large enough [5]. In males, the ectopic ureter can return to the urethra via the seminal vesicles, the deferential duct, or the epididymis. Moreover, the fertility status should always be investigated in patients with ZS since it has been associated with infertility in up to 45% of the cases.

ZS is linked to other anomalies, including megaureter and ectopic ureter. The Wolffian (mesonephric) duct is a paired organ during embryogenesis. The incidence of renal agenesis or dysplasia depends on the timing of the insult during embryogenesis before seven weeks of gestation (before the ureteric bud appears). The maldevelopment of the mesonephric duct (distal portion) results in ejaculatory duct atresia, which obstructs and collects secretion in the seminal vesicle, causing cysts. The developmental pathology causes azoospermia or oligozoospermia to manifest as primary infertility and pelvic and perineal pain. ZS is considered the male counterpart of Mayer-Rokitansky-Kustner-Hauser (MRKH) syndrome in females. The ureteric bud distal to the urogenital sinus has insufficient time to become independent from the mesonephric duct. The removal of the ureteric bud from its migration outside the primitive urinary bladder results in the formation of an ectopic ureter.

The underlying pathophysiology of ZS is still mostly unknown. One plausible hypothesis is that, even in cases where the contralateral testis is unobstructed, the unilateral testicular blockage could lead to the generation of anti-sperm antibodies and infertility [6]. Cito et al. [7] suggested that reactive oxygen species may cause reproductive damage in ZS patients due to persistent blockage, lowering the sperm count through germ cell apoptosis.

The clinical findings indicate that individuals with ZS are asymptomatic until their second to fifth decade of life and that symptoms typically manifest during intense sexual or reproductive activity. Depending on the seminal vesicle cyst's size, which is less than 5 cm in diameter, they are commonly asymptomatic [8]. Due to lack of non-specific symptoms of ZS, it remains undiagnosed or misinterpreted. ZS is incidentally diagnosed during the digital rectal examination or cross-sectional imaging. Due to ejaculatory duct obstruction, ZS is characterized by a low ejaculatory volume of sperm, oligozoospermia, and eventually azoospermia. An alkaline pH, a high citrate level, and a lower concentration of fructose and carnitine in the seminal plasma can cause infertility in up to 45% of the cases [9]. Sometimes, ejaculatory disorders cause painful ejaculation and hematospermia. Other signs of ZS are usually non-specific signs related to urination, such as prostatism, urinary urgency, frequency, dysuria, hematuria, pelvic/perineal discomfort, and scrotal, hypogastric, or defecation pain. The symptoms of ZS can lead to complications such as chronic recurrent epididymitis or prostatitis. Seminal vesicle cysts are more significant than 12 cm and are called giant cysts, which are also likely to cause urinary bladder obstruction, triggering lower urinary tract infections and colonic obstruction. Malignant transformation of the seminal vesicle cyst is rare.

Pavan et al. [10] reported a large para-testicular mass mimicking a varicocele. Van den Ouden et al. [11] correlated ZS with the following symptoms: dysuria (37%), frequency (33%), perineal pain (29%), epididymitis (29%), and irritation following ejaculation (21%).

Imaging remains the key to the diagnosis of ZS. Today's imaging techniques offer many modalities for evaluating and distinguishing seminal vesicle cysts from other pelvic masses.

The most commonly used non-invasive technique is ultrasonography (USG) to evaluate patients with suspected development failure of the mesonephric duct [12]. Typically, transrectal or abdominal ultrasound is the initial method for diagnosing and assessing pelvic masses. Transabdominal and transrectal USG show that a seminal vesicle cyst is a silent pelvic mass thought to be an extrinsic smooth-walled filling defect along the inferolateral bladder surface with a thick wall that may or may not show mural calcification. Infection, or bleeding from the cyst may appear as a cystic mass containing internal echoes.

The most successful diagnostic technique is transrectal ultrasonography (TRUS) with seminal vesicle aspiration, as demonstrated by Avellino et al. in their review [13]. Vasovesiculography shows an enlarged vesicle, a misshaped seminal vesicle, a blocked ejaculatory duct, and the backflow of contrast into an ipsilateral ectopic ureter.

CT and MRI are the most precise imaging modalities for the evaluation of the genitourinary system and can provide the information necessary to confirm ZS without additional invasive investigations. ZS is characterized by a well-defined retrovesicular cystic pelvic mass of water or near-water attenuation or seminal vesicle cyst with a thick irregular wall or hyperdense contents seen just above the prostate gland, with ipsilateral seminal vesicle enlargement associated with ipsilateral renal agenesis, which may not be enough to confirm the diagnosis.

MRI is considered the "gold standard" in the diagnosis and surgical planning of ZS or mesonephric duct malformation because it can be used in multiple planes, has excellent soft tissue resolution, and doesn't use ionizing radiation. MRI images provide a more accurate description of the cyst's contents, accurate detection of associated genitourinary abnormalities, and an assessment of the anatomic relationship between the pelvic structures. Tubular and multilocular cystic masses are less common than unilocular masses. MRI scans reveal the seminal vesicle cyst appearing hypointense on T1-weighted images and intense on T2-weighted images. Protein-rich contents or a previous episode of bleeding inside the cyst cause strong or weak signals on T1- and T2-weighted images [14]. Malignancy is indicated by intra-cystic vegetation, but malignant degeneration is rare, with only three cases reported in the literature. MRI can comprehensively evaluate all pelvic organs and characterize masses using post-contrast graphic sequences. Not only does this result in a definitive diagnosis, but the imaging may also be helpful for the surgical planning of seminal vesicle cyst excision, as it reveals the anatomical relationships between the pelvic structures. Occasionally, MRI can reveal the connection between the ectopic ureter and seminal vesicle, which is overlooked during investigation. The location of the cysts (median, paramedian, or lateral), developmental issues like renal agenesis issues with the external genitalia, or how they appear on an MRI can all help distinguish between different cyst types. Urography of the excretory system can show ipsilateral renal agenesis and an abnormal part of the collecting system. Urography of the excretory system can reveal associated ipsilateral renal agenesis or dysgenesis.

Engin et al. [15] noted that TRUS was an excellent way to diagnose endorectal MRI-associated obstruction (EDO), particularly when there was complete obstruction. MRI, however, is helpful in examining soft tissue and cystic lesions. Seminal vesicle cysts are a differential diagnosis for several pelvic organ cystic diseases. Examples are true prostate gland cysts, ureteroceles, Müllerian duct cysts, and Gartner's duct cyst.

The management of ZS depends on the size, morphology, symptomatology, and complications of seminal vesicle cysts. For individuals with ZS with no symptoms or minimal symptoms, treatment is usually conservative and includes antibiotics, percutaneous drainage, or transurethral or transrectal aspiration of the seminal vesicle cyst; both are associated with a greater risk of recurrence. The treatment for individuals with ZS who failed conservative treatment or have symptomatic cysts larger than 5 cm includes invasive or surgical procedures like open or minimally invasive surgery, laparoscopic transperitoneal approaches, or robotic-assisted approaches because they have the advantages of a direct approach, less invasiveness, magnification, and good visualization [16]. 

In 2003, Valla et al. [17] sought to maintain fertility by laparoscopically excising growing seminal cysts from a 15-month-old kid. Over 20 months, the cystic tumor, which was detected during pregnancy, grew in size from 12 to 25 mm. Differential diagnoses of ZS include ureterocele, prostatic utricle cysts, acquired seminal vesical cysts, and ejaculatory duct cysts and are ruled out based on clinical and imaging findings.

Review of literature

A review of the published literature on ZS was made and has been compiled in the form of a table (Table 1).

**Table 1:** The authors, year of publication, key findings, methodology, sample size, and outcomes from previously published literature on Zinner syndrome MRI: magnetic resonance imaging ZS: Zinner syndrome

Author(s)	Year	Key Findings	Methodology	Sample Size	Outcome
Mehra S et al., 2016 [1]	2016	ZS diagnosed on MRI	Case report	1	Highlighted the role of MRI in diagnosing ZS
Zinner A 1914 [2]	1914	First description of ZS	Case report	1	Introduced the syndrome
Liu T et al., 2021 [3]	2021	Pooled analysis of ZS cases	Systematic review	214	Updated analysis on ZS
Sheih CP et al., 1990 [4]	1990	Link between pelvic dilatations and ipsilateral renal agenesis	Study	N/A	Identified a rare frequency of ZS
Kao CC et al., 2010 [5]	2010	Case report on ZS mimicking bladder outlet obstruction	Case report	1	Demonstrated the diagnostic challenge of ZS
Pereira BJ et al., 2009 [6]	2009	Review based on a clinical case	Review	1	Emphasized clinical presentation and literature review
Cito G et al., 2019 [7]	2019	Infertility in ZS and the role of seminal tract obstruction	Case presentation	1	Discussed reproductive damage from ZS
Van den Ouden D et al., 1998 [11]	1998	Management of seminal vesicle cysts in ZS	Pooled analysis	52	Offered insights into symptoms and management

## Conclusions

Zinner syndrome, a rare congenital anomaly characterized by renal agenesis, seminal vesicle cysts, and ejaculatory duct anomalies, poses diagnostic challenges due to its subtle clinical presentation. While some patients remain asymptomatic, others experience urinary tract infections, pain, and fertility issues. Diagnosis often relies on MRI imaging for a detailed assessment. Management strategies, ranging from conservative to surgical interventions, depend on symptom severity and fertility desires. However, there is a notable gap in understanding long-term outcomes, necessitating further research to improve patient care. Increased awareness among healthcare providers and individualized management plans are crucial for optimizing outcomes in the affected individuals.
